# Genetic and Morphological Variation Among Populations of Duckweed Species in Thailand

**DOI:** 10.3390/plants14132030

**Published:** 2025-07-02

**Authors:** Athita Senayai, Yosapol Harnvanichvech, Srunya Vajrodaya, Tokitaka Oyama, Ekaphan Kraichak

**Affiliations:** 1 Department of Botany, Faculty of Science, Kasetsart University, Bangkok 10900, Thailand; athita.s@ku.th (A.S.); yosapol.harn@ku.th (Y.H.); srunya.v@ku.th (S.V.); 2 Department of Botany, Graduate School of Science, Kyoto University, Kyoto 606-8502, Japan; oyama.tokitaka.8w@kyoto-u.ac.jp; 3 Duckweed Holobiont Resource & Research Center (DHbRC), Kasetsart University, Bangkok 10900, Thailand; 4 Biodiversity Center, Kasetsart University (BDCKU), Bangkok 10900, Thailand

**Keywords:** duckweed, genetic variation, morphological variation, MLST

## Abstract

Duckweeds have emerged as frontier plants in research, food, and bioenergy applications. Consistency in genetic and morphological traits within species is therefore crucial for their effective use. Thailand hosts diverse duckweed populations with representatives from four of the five genera and at least four species recorded. However, the extent of genetic and morphological variation within these species in Thailand remains unclear. Here, we investigated the genetic and morphological variation in four duckweed species—*Landoltia punctata*, *Lemna aequinoctialis*, *Spirodela polyrhiza*, and *Wolffia globosa*—collected from 26 sites across Thailand. Using the multilocus sequence typing approach based on three chloroplast genes (*rbcL*, *atpF–atpH*, and *psbK–psbI*), we show that genetic variation in duckweed is distinct at both inter-species and intra-species levels. Among these four species, *Lemna aequinoctialis* exhibits the highest genetic variation, forming four distinct phylogenetic clusters. This is followed by *Spirodela polyrhiza*, *Wolffia globosa*, and *Landoltia punctata*. In addition, we observe that morphological variation, particularly frond aspect ratio, varies significantly among clusters but remains consistent within each cluster of each species. These findings suggest that duckweed populations in Thailand exhibit substantial genetic variation at the intraspecific level, which is closely associated with frond morphological variation.

## 1. Introduction

Duckweeds are free-floating aquatic angiosperms classified under the family Lemnaceae [1,2,3,4]. This family comprises 35 species and two interspecific hybrid species across five genera worldwide: *Landoltia*, *Lemna*, *Spirodela*, *Wolffia*, and *Wolffiella* [5,6]. Of these, *Landoltia, Lemna, Spirodela*, and *Wolffia* have been found in Thailand. Duckweeds are known for their rapid vegetative reproduction and remarkable growth rates, with the ability to double their biomass within 1–3 days. For instance, *Wolffia microscopica* has been reported to double its biomass in a day [7]. This rapid growth makes duckweeds an attractive resource for bioethanol fermentation and bioenergy applications [8]. Beyond their productivity, duckweeds are nutritionally rich, with protein content of up to 35% by weight depending on species and growth conditions, along with essential vitamins [9,10,11]. These nutritional benefits support their application in livestock feed and point to their potential as a sustainable food source for humans [11,12,13,14,15,16]. In Thailand, duckweeds are commonly found in freshwater ecosystems and have traditionally been used as animal feed. Among them, *Wolffia globosa*, locally known as *khai-nam* or *khai-pham*, has a long history of culinary use, particularly in the Northern and Northeastern Thai regions. With duckweeds gaining recognition for their diverse applications, understanding their genetic variation is a key for identifying suitable strains for targeted use.

Genetic and morphological variations among duckweed species significantly impact their application potential. At the interspecies level, duckweeds exhibit variation in biochemical traits, such as fatty acid composition. For instance, *Wolffia borealis* contains fatty acids up to 14%, whereas *Wolffiella welwitchii* contains only 4% [17]. Substantial intraspecies variation has also been documented, particularly in starch content and protein. For instance, a study on *Lemna minor* reported starch content ranging from 26.3% to 45.5% of dry weight [18]. In *Landoltia punctata,* protein content has been reported to range from 20% to 35% [19]. These findings emphasize the critical role of screening genetic and morphological variation to identify duckweed strains and maximize their utility in applications [20].

The genetic variation in duckweed has been shown to be influenced by geographical factors. Distinct geographical patterns have been observed in duckweed populations across regions such as Asia, Europe, and North America. For instance, global sampling of *Spirodela polyrhiza* revealed four major genetic clusters corresponding to geographic origins—America, Europe, India, and Southeast Asia—demonstrating the influence of regional separation on genetic structure [21]. Similarly, studies in China have shown that populations of *Lemna aequinoctialis* and *Wolffia globosa* exhibit genetic diversity across different regions, likely shaped by geographical factors [22,23]. However, despite duckweeds being abundant in Thailand, a comprehensive study of genetic variation within the country has yet to be conducted.

Here, we investigate the genetic and morphological diversity of four duckweed species in Thailand—*Landoltia punctata*, *Lemna aequinoctialis*, *Spirodela polyrhiza*, and *Wolffia globosa*—collected from 26 sites across the country. Phylogenetic and multilocus sequence typing analyses of chloroplast markers verified species identities and intraspecific genetic variation in Thai species. To complement the genetic data, we assess frond morphology across populations and explore its association with genetic patterns. Together, these findings offer the first comprehensive overview of duckweed diversity in Thailand and highlight the potential of locally adapted strains for future applications.

## 2. Results

### 2.1. Species Identification of Thai Duckweeds

**Figure 1:**
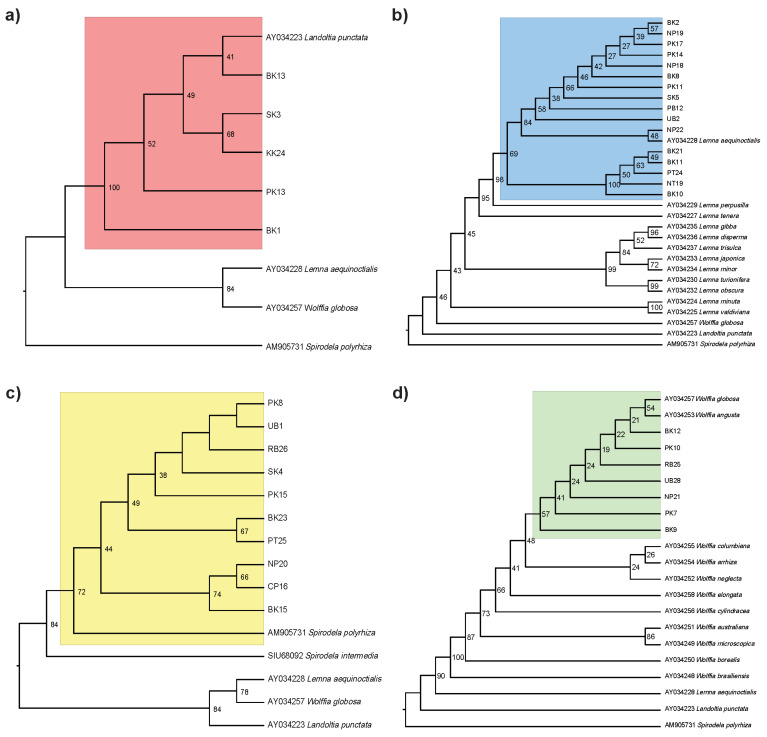
Phylogenetic relationships within four duckweed genera based on Thai duckweed samples. Maximum likelihood trees were constructed using *rbcL* gene sequences for (**a**) *Landoltia*, (**b**) *Lemna*, (**c**) *Spirodela*, and (**d**) *Wolffia*. Thai duckweed accessions are labeled with alphanumeric codes (e.g., BK13, NP19), while reference sequences obtained from NCBI are annotated with species names and corresponding GenBank accession numbers.

To verify the species’ identity of duckweed samples across Thailand, we identified the species using morphological characters and extracted their DNA to obtain the chloroplast-encoded *rbcL* sequence data. The maximum likelihood analysis of *rbcL* sequences confirmed genus-level identification across duckweed samples (Figure 1; Table S1). All five *Landoltia* samples formed a strongly supported monophyletic clade with the reference *La. punctata* (AY034223; bootstrap support = 100). Sixteen *Lemna* samples clustered with *Le. aequinoctialis* (AY034228; bootstrap support = 69). The ten *Spirodela* samples formed a monophyletic group with the *S. polyrhiza* reference (AM905731) with bootstrap support of 72. This clade was clearly separated from its sister species, *S. intermedia*, supporting its recognition as a distinct species. Seven *Wolffia* samples formed a weakly supported grade (bootstrap support = 57) with *W. globosa* (AY034257) and *W. angusta* (AY034253). Notably, the *rbcL* sequences from our *Wolffia* samples formed a sister clade to both *W. globosa* and *W. angusta*, indicating their limited phylogenetic divergence among *Wolffia* species.

### 2.2. Genetic Diversity of Thai Duckweeds

To assess intraspecific genetic diversity of duckweeds in Thailand, sequences from three chloroplast markers (*rbcL*, *atpF-atpH*, and *psbK-psbI*) were concatenated for each sample and analyzed for haplotype diversity (h) and nucleotide diversity (π). The resulting concatenated sequence lengths ranged from 1340 bp in *W. globosa* to 1484 bp in *S. polyrhiza*, with a relatively consistent GC content of 33.19–34.00% (Table 1). Haplotype diversity analysis revealed a maximum value (h = 1.000) in all species, indicating high genetic variability at the intraspecific level (Table 1). In addition, nucleotide diversity varied considerably among species, with *W. globosa* showing the highest value (π = 0.099) and *S. polyrhiza* the lowest (π = 0.032), indicating different levels of intraspecific genetic variation (Table 1). The number of segregating sites (S) across four duckweed genera ranged from 152 in *La. punctata* to 502 in *Le. aequinoctialis*. To further assess nucleotide diversity and test whether nucleotide variation deviates from neutral evolution, we calculated Tajima’s D, which compares the number of segregating sites to the average nucleotide differences. All species showed negative Tajima’s D values, with significant results in *S. polyrhiza* (D = −3.344, *p* = 0.001) and *Le. aequinoctialis* (D = −2.276, *p* = 0.023). These negative values could result from multiple demographic and evolutionary processes, including purifying selection, recent population expansion, or population structure effects where pooling individuals from genetically differentiated populations enriches the dataset with rare alleles (Table 1). In contrast, populations of *La. punctata* (D = −1.625, *p* = 0.104) and *W. globosa* (D = −1.271, *p* = 0.204) were more consistent with neutral evolution, indicating more stable populations throughout Thailand (Table 1).

### 2.3. Population Structures of Thai Duckweeds

**Figure 2:**
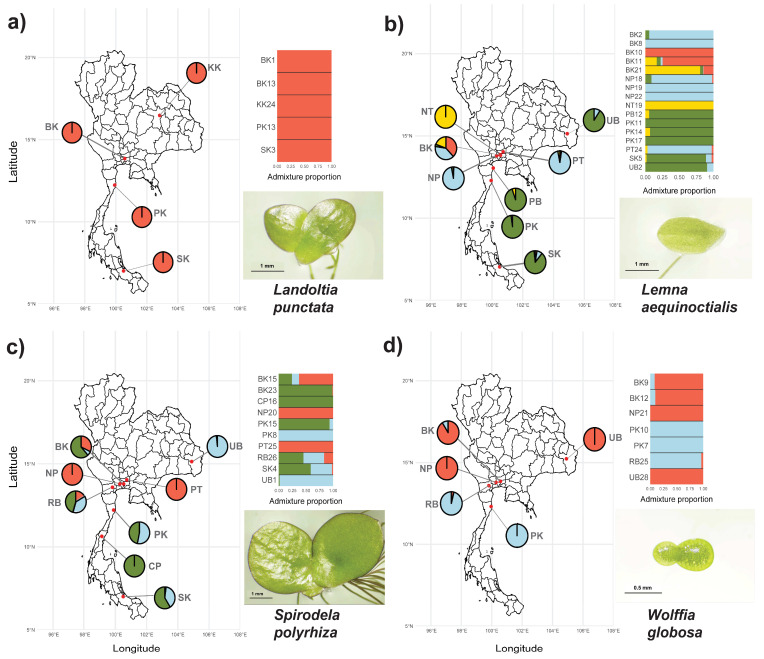
Geographical distribution and admixture proportions of four duckweed species across Thailand. Maps show sampling locations overlaid on provincial boundaries, with pie charts indicating admixture coefficients derived from population structure analysis. Adjacent bar plots represent admixture proportions for individual accessions within each population. The studied species include (**a**) *Landoltia punctata*, (**b**) *Lemna aequinoctialis*, (**c**) *Spirodela polyrhiza*, and (**d**) *Wolffia globosa*. Inset images highlight representative frond morphology for each species. Scale bars: 1 mm (**a**–**c**), 0.5 mm (**d**).

To assess the genetic structure and potential gene flow among Thai duckweed populations, we conducted admixture analysis using concatenated chloroplast markers (*rbcL*, *atpF-H*, and *psbK-I*). The analysis revealed distinct population structures across the four studied species, with the optimal number of genetic clusters (K) varying from 1 to 4 (Figure 2).

*Landoltia punctata* showed no population structure (K = 1) across five populations, consistent with its low number of segregating sites (S = 152) and non-significant Tajima’s D, indicating genetic homogeneity (Figure 2a). In contrast, *Le. aequinoctialis* exhibited the highest genetic complexity (K = 4) among 16 populations, corresponding to high polymorphism (S = 502) and a significantly negative Tajima’s D (D = −2.276, *p* = 0.023), with some clusters showing geographical patterns across Thailand (Figure 2b). *Spirodela polyrhiza* formed three clusters (K = 3) across 10 populations, aligned with its high genetic variation (S = 452) and strongly negative Tajima’s D (D = −3.344, *p* = 0.001). Although clusters were broadly distributed, one cluster was predominantly found in central Thailand (Figure 2c). *Wolffia globosa* displayed two clusters (K = 2) among seven populations, despite its relatively high nucleotide diversity (π = 0.099). These clusters showed moderate geographic structuring, with both clusters co-occurring in central Thailand (Figure 2d). These findings demonstrate varying levels of genetic differentiation and population structure among Thai duckweed species, suggesting species-specific patterns of diversity and regional distribution.

### 2.4. Morphological Variation: Univariate Analysis

**Figure 3:**
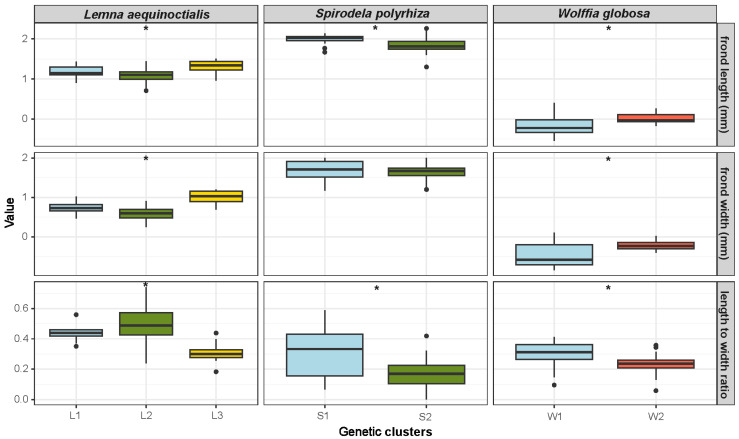
Natural log-scale variation in morphology across genetic clusters in three duckweed species. Box plots display the distribution of log-transformed values for frond length, frond width, and length-to-width ratio among genetic clusters of *Lemna aequinoctialis* (L1–L3), *Spirodela polyrhiza* (S1–S2), and *Wolffia globosa* (W1–W2). Columns represent species, while rows correspond to the three morphological traits. Morphometric differences among genetic clusters illustrate the extent of trait divergence within each species. Asterisks indicate significant differences between genetic clusters within each species based on non-parametric tests (* *p* < 0.01).

To assess whether genetic variation among population clusters is associated with any specific morphological traits, we further analyzed frond morphology from fresh specimens—including frond length, width, and aspect ratio—in three duckweed species exhibiting population structure: *Le. aequinoctialis*, *S. polyrhiza*, and *W. globosa* (Figure 3, Table S2). Individuals from BK10, BK11, PT25, and NP20 were not available as fresh specimens. Therefore, their respective genetic clusters were not included in the subsequent morphological analysis, reducing the number of genetic clusters to three for *Le. aequinoctialis* and two for *S. polyrhiza*.

In *Le. aequinoctialis*, all measured morphological traits showed highly significant differences among genetic clusters (*p* < 0.001). Cluster L3 individuals had significantly longer fronds than Clusters L1 and L2 (*p* < 0.001). Frond width also differed significantly across all pairwise comparisons (*p* < 0.001), with Cluster L3 having the widest fronds, followed by L1 and L2. The length-to-width ratio also varied significantly among clusters (*p* < 0.001), with Clusters L1 and L2 displaying higher ratios than L3.

In *S. polyrhiza*, Cluster S1 exhibited longer fronds and a higher length-to-width ratio than those of S2 (p_length_ < 0.001, p_ratio_ < 0.01). However, frond width did not differ significantly between clusters (*p* = 0.69).

In *W. globosa*, the analysis revealed significant differences between the two genetic clusters in all morphological traits (*p* < 0.01). Cluster W2 exhibited larger dimensions in both frond length and width compared to W1. However, Cluster W1 showed a higher length-to-width ratio than W2. These results suggested morphological differentiation among the observed genetic groups in the three Thai duckweed species.

### 2.5. Morphological Variation: Bivariate Analysis

**Figure 4:**
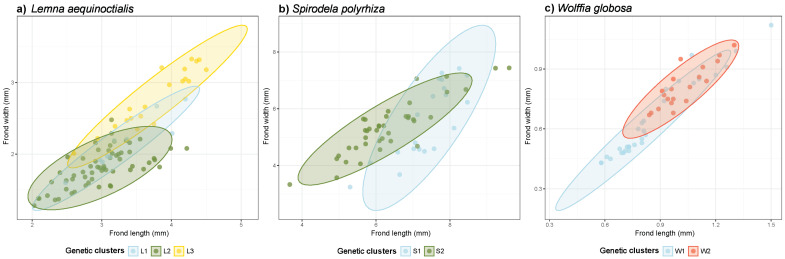
Scatter plots depicting frond length and frond width in three duckweed species: (**a**) *Lemna aequinoctialis*, (**b**) *Spirodela polyrhiza*, and (**c**) *Wolffia globosa*. Each point represents an individual frond with 95% confidence ellipses showing cluster distributions, color-coded by genetic cluster (L1–L3, S1–S2, and W1–W2 for the respective species).

To investigate whether genetic clustering is associated with bivariate morphological variation, we drew scatter plots using frond length and width across three duckweed species. The bivariate plots revealed morphological differentiation among genetic clusters in all three species (Figure 4).

In *Le. aequinoctialis*, the scatter plot revealed distinct morphological clustering that corresponded with the three genetic clusters (L1, L2, and L3) (Figure 4a). Cluster L3 (yellow) had the largest fronds and occupied a distinct region in the morphospace, while clusters L1 (blue) and L2 (green) showed considerable overlap in the smaller size range.

In *S. polyrhiza*, two genetic clusters (S1 and S2) were separated in the morphospace (Figure 4b). Cluster S1 (blue) contained large-frond individuals that formed a relatively compact group in the upper size range. Cluster S2 (green) exhibited a broader distribution across smaller sizes, indicating greater morphological variability in this cluster. 

In *W. globosa*, the scatter plot revealed that individuals within genetic clusters exhibited some overlap in their morphological space (Figure 4c). Cluster W1 (blue) occupied the smaller size range, while cluster W2 (red) showed slightly larger fronds but with substantial overlap between clusters. Overall, the bivariate analysis indicated that morphological differences could be found among the genetic clusters within each of the three species.

## 3. Discussion

### 3.1. Phylogenetic Placement of Thai Duckweeds

The phylogenetic reconstruction of duckweed sequences identified Thai duckweed samples as *Landoltia punctata*, *Lemna aequinoctialis*, *Spirodela polyrhiza*, and *Wolffia globosa*. Our findings demonstrated that the *rbcL* marker was efficient at discriminating species of Thai duckweed, despite its suboptimal performance at the global scale [24]. The only exception was the placement of *W. globosa* with *W. angusta*. However, the distributions of these two *Wolffia* species are distinct. *W. globosa* has been documented in Thailand [25], whereas *W. angusta* has been reported in Australia [16]. While a multilocus dataset would yield more precise species identification, sequences of multiple markers (especially of *atpF-H* and *psbK-I*) were not consistently available in the database for the same voucher specimens, preventing us from using this approach [26]. Therefore, only the *rbcL* sequence data were used to determine the phylogenetic placement of Thai specimens.

Our phylogenetic reconstruction revealed relationships between some Thai species and morphologically similar species that were often mistaken for each other. The first pair was between *Le. aequinoctialis* and *Le. perpusilla*, which have different distributions. *Lemna aequinoctialis* is considered a cosmopolitan species, while *Le. perpusilla* has been reported to have a distribution limited to North America [16]. Similarly, confusion may arise between *W. globosa* and *W. arrhiza*. One of the earliest reports for *Wolffia* in Thailand identified the local species as *W. arrhiza* [27]. However, later studies by taxonomists and other duckweed biologists confirmed the species in Thailand as *W. globosa* [25], while *W. arrhiza* is mainly distributed in South Africa and Europe [16]. The use of molecular markers in conjunction with morphological determination can be more effective in distinguishing plants in this family in Thailand.

### 3.2. Genetic Diversity and Population Structures of Thai Duckweeds

The study of four duckweed species from Thailand revealed genetic diversity and distinct population structure within these species. The observed pattern of genetic diversity showed that *W. globosa* exhibited the highest genetic diversity, followed by *Le. aequinoctialis*, while *S. polyrhiza* and *La. punctata* showed the lowest genetic diversity. This pattern is consistent with previous findings in Chinese duckweed populations [28], suggesting conserved genetic diversity patterns and reproductive biology of each species across different geographical regions.

*Wolffia globosa* exhibited the highest nucleotide diversity (π = 0.099) but only two genetic clusters. Two distinct geographic patterns were found in this species. While the clustering of populations in Bangkok and neighboring Nakhon Pathom and Ratchaburi provinces showed evidence of local gene flow, the distant areas from the central region revealed a homogeneous cluster in Northeastern (Ubon Ratchathani) and Southwestern (Prachuap Khiri Khan) areas, which were geographically diverse from each other, suggesting efficient long-distance dispersal of the genetic clusters. The non-significant Tajima’s D indicated a more stable long-term population history of the species, contrasting with the high genetic diversity. This pattern might result from long-term persistence of distinct lineages with recent admixture events, possibly facilitated by human activities or changes in habitat connectivity. As this species has traditionally been consumed as food by Thai people in the North and Northeastern regions, it is now gaining increased recognition as a health-promoting food source [29]. The potential for gene flow may be attributed to the growing trend of individuals purchasing the plant online for cultivation, either for commercial purposes or personal consumption.

Among Thai duckweed species, *Le. aequinoctialis* showed the highest genetic diversity (S = 502, π = 0.048) and the most complex population structure with four genetic clusters. The geographic distribution of these clusters revealed intriguing patterns. Central Thailand appeared to be the center of genetic diversity, as three of the four clusters were found in this region. Bangkok populations included the members from all four diverse genetic clusters. Although some duckweed populations in Bangkok showed barriers to gene flow despite their proximity (such as BK8 and BK10), others exhibited evidence of gene flow between them (BK2 and BK8). Intriguingly, genetic similarity was found between long-distance areas, including the Northeastern (Ubon Ratchathani), Southwestern (Phetchaburi and Prachuap Khiri Khan), and Southern (Songkhla) populations. This suggested that this species might have efficient natural dispersal mechanisms or be unintentionally dispersed by human activities, such as aquaculture and fish transportation. The significant negative Tajima’s D (D = −2.276, *p* = 0.023) could result from purifying selection, recent population expansion, or population structure effects. Given the four distinct genetic clusters observed, pooling genetically differentiated populations likely contributed to this pattern by enriching rare alleles in the dataset. This high genetic diversity and complex population structure may also be attributed to hybridization events within this species complex. Recent studies have revealed that *Le. aequinoctialis* represents a taxonomically complex group shaped by extensive interspecific hybridization and polyploidy [30]. Their genomic analysis demonstrated recurrent hybridization events, including the presence of autotriploid clones within *Le. aequinoctialis* and tetraploid hybrids between *Le. aequinoctialis* and *Le. perpusilla*, with these fertile tetraploid hybrids being capable of further reproduction and dispersal across regions. 

*Spirodela polyrhiza* showed a moderate level of nucleotide diversity (π = 0.032) compared to the other species. While we observed low nucleotide diversity, consistent with findings by Bog et al. [31] and Xu et al. [21], we also found a high number of segregating sites (452) and a highly significant negative Tajima’s D (D = −3.344, *p* = 0.001). This suggested that populations of *S. polyrhiza* in Thailand may be undergoing recent demographic changes or selective pressures not observed in other studied populations. The population structure of this species showed distinct genetic clusters of duckweed populations in the central region, with a genetic cluster (red) in Nakhon Pathom and Pathum Thani that might be a core cluster for this genetic cluster or have effective adaptation to the environment in this area, similarly to *Le. aequinoctialis* in these two provinces. Bangkok and surrounding central provinces appear to be significant areas for both *Le. aequinoctialis* and *S. polyrhiza* genetic diversity, as evidenced by high segregating sites and mixed genetic clusters in their populations. The significant negative Tajima’s D values could result from multiple processes, including recent population expansion, demographic changes, or population structure effects from pooling genetically differentiated populations. For both species, this pattern may reflect human-mediated dispersal through aquaculture and fish transportation activities in this region. Additionally, for *Le. aequinoctialis*, hybridization events may contribute to the observed genetic complexity. Furthermore, the significant negative Tajima’s D values observed in our study for *Le. aequinoctialis* and *S. polyrhiza* were in interesting contrast with the findings of Tang et al. [32] in Lake Tai, China, where positive Tajima’s D values were observed. However, the difference in genes used between our study and Tang et al. [32] may cause the differentiation of intraspecific distance [24], and the different approaches may be causing the different interpretations. This finding highlighted the importance of regional studies in understanding duckweed population dynamics, specifically in specific regions.

*Landoltia punctata* exhibited the lowest genetic diversity (S = 152, π = 0.038) among the studied species. This finding aligned with global trends of low genetic diversity in *La. punctata*, as reported by Tang et al. [32] in Chinese populations and Bog et al. [31] in global samples. An allozyme study of *La. punctata* revealed that eight out of fourteen multilocus genotypes came from Australia and Southeast Asia, suggesting a hotspot for genetic diversity [33]. However, this study included only one sample from Central Thailand. The observed genetic diversity in allozymes might therefore reflect differences between broader Southeast Asian populations rather than within Thailand itself.

Analysis of frond morphology in *Le. aequinoctialis*, *S. polyrhiza*, and *W. globosa* revealed overall significant differences in most morphological traits among the genetic clusters of each species. These results suggested that morphological variation is substantially influenced by genetic factors, likely resulting from long-term evolutionary processes and selective pressures. Phenotypic plasticity in response to environmental factors appears to play a less significant role in shaping frond morphology among duckweed populations in Thailand, although the varying degrees of morphological overlap between some genetic clusters suggest some environmental influence. These findings highlight the need for further ecological studies to precisely assess how genetics and environment influence the morphological traits of duckweeds. Moreover, this study provides a foundation for investigating other characteristics that could lead to applications of these plants, such as physiological and phytochemical characteristics. Such investigations may reveal additional trait differences that could be useful for strain selection of Thai duckweeds with potential applications in product development or as nutritionally enhanced food sources, ultimately improving the economic viability of these plants in Thailand.

## 4. Materials and Methods

### 4.1. Sampling Sites and Sample Preparation

Thirty-eight samples of duckweeds from 26 study sites across Thailand were collected during July 2020–April 2021 (Table S3). All duckweed samples were identified to the species according to their morphology using the key and descriptions in the Flora of Thailand (Lemnaceae) and other relevant taxonomic literature [25,34]. Samples were surface sterilized by soaking in 1.5% sodium hypochlorite (NaClO) for 30 s, followed by three rinses in distilled water. The remaining surface contaminants were carefully removed under a stereomicroscope using fine brushes and further cleaned using a sonicator. Fresh tissues were then frozen at −20 °C for subsequent DNA extraction.

### 4.2. DNA Extraction, PCR Amplification, and Sequencing

DNA from duckweed samples was extracted using the Nucleospin^®^ Plant II Kit (Macherey-Nagel GmbH & Co., Düren, Germany). Three chloroplast DNA markers, including *rbcL*, *atpF-H*, and *psbK-I*, were amplified using specific primer sets (Table S4). PCR amplification of the *rbcL* gene was performed under the following conditions: initial denaturation at 94 °C for 4 min, followed by 30 cycles of denaturation at 94 °C for 1 min, annealing at 51 °C for 50 s, and extension at 72 °C for 1.5 min, with a final extension at 72 °C for 10 min. For the markers with non-coding spacers (*atpF-H* and *psbK-I*), PCR conditions were initial denaturation at 94 °C for 4 min, followed by 35 cycles of denaturation at 94 °C for 30 s, annealing at 50 °C for 30 s, and extension at 72 °C for 1 min, with a final extension at 72 °C for 10 min. PCR products were visualized on 1.0% agarose gels in 1X Tris-Borate-EDTA buffer. Successfully amplified products were purified using ExoSAP-IT™ (Thermo Fisher Scientific, Waltham, MA, USA). Purified PCR products were then sent to Macrogen Inc. (Seoul, Republic of Korea) for bidirectional Sanger sequencing.

### 4.3. Phylogenetic Analysis

Phylogenetic relationships within each duckweed genus were reconstructed using *rbcL* gene sequences to complement morphological identification of Thai duckweed species. The *rbcL* marker is widely used as a standard DNA barcode for flowering plants [35]. Samples representing four genera (*Landoltia*, *Lemna*, *Spirodela*, and *Wolffia*) were analyzed with additional sequences from the National Center for Biotechnology Information (NCBI). The reference sequences included *Landoltia punctata* (accession: AY034223), *Lemna aequinoctialis* (accession: AY034228), *Spirodela polyrhiza* (accession: AM905731), and *Wolffia globosa* (accession: AY034257), for their respective genera. Additional species within each genus were also included in the analysis (Table S5). Outgroup species from other duckweed genera were used to ensure robust phylogenetic reconstruction (Table S5). The *rbcL* sequences were aligned using MAFFT v7.511 with the L-INS-i method [36]. Alignment was subsequently refined using Gblocks v0.91b [37] with the following parameters: the minimum number of sequences for a flank position was set to 70% of the total sequence number, and gap positions were allowed if present in less than 50% of the sequences. All other Gblocks parameters were kept at default values. Phylogenetic analysis was conducted for each duckweed genus using multiple samples alongside selected reference sequences. Phylogenetic trees were reconstructed using the Maximum-Likelihood method implemented in IQ-TREE v2.3.2 [38], with node support evaluated through 1000 bootstrap replicates, and visualized using FigTree v.1.4.4 [39].

### 4.4. Population Structure and Genetic Data Analysis

DNA sequences from *rbcL*, *atpF-atpH*, and *psbK-psbI* regions were aligned separately using MAFFT v7.511 with the L-INS-i method [36]. Alignments were further refined using Gblocks 0.91b [37] with the following parameters: the minimum number of sequences for a flank position was set to 70% of the total sequence number, and gap positions were allowed if present in fewer than half of the sequences. All other Gblocks parameters were maintained at their default settings. The refined alignments of the three markers for each species were concatenated using MEGA11 (version 11.0.13) [40] for subsequent genetic diversity analyses. Population structure of each species was analyzed using an admixture model-based approach [41] implemented in R statistical software (version 4.1.2) [42]. This model allows for mixed ancestry, where individuals can have genetic contributions from multiple populations (K). The optimal number of clusters (K) was determined using the cross-entropy criterion, with lower values indicating better model fit [43]. Genetic diversity parameters included GC content (%), haplotype diversity (h), number of segregating sites (S), nucleotide diversity (π), and Tajima’s D. The analysis utilized packages adegenet, ape, pegas, poppr, tidyverse, and LEA [44,45,46,47,48,49].

### 4.5. Morphological Study and Analysis

Twenty representative populations from different genetic clusters of three duckweed species—*Le. aequinoctialis*, *S. polyrhiza*, and *W. globosa*—were selected for frond morphological analysis. Fresh specimens were collected directly from natural habitats during the field sampling period (July 2020–April 2021) and maintained in their original water samples. To determine the optimal sample size, the coefficient of variation (CV) was assessed using increasing sample sizes, with CV values stabilizing at ten fronds per population (CV range: 5.0–18.7%), indicating that additional measurements beyond this number would not significantly improve the precision of morphological characterization. Therefore, ten fully expanded fronds per population were selected from field-collected material, ensuring that fronds showed no signs of damage, disease, or senescence.

Morphological analysis was conducted within 48 h of collection to preserve natural characteristics. Since multiple duckweed species often co-occurred in the same water bodies, individual fronds were first carefully sorted by species using a fine brush under magnification to ensure taxonomic accuracy prior to both morphological analysis and DNA extraction. Selected fronds were then transferred to Petri dishes containing tap water for imaging. Specimens were photographed using a Motic^®^ SMZ-171 stereomicroscope (Motic Microscope, Universal City, TX, USA) equipped with a Canon EOS800D digital camera (24.2 megapixel CMOS sensor, Canon Inc., Tokyo, Japan). All images were captured at consistent resolution (6000 × 4000 pixels, 72 dpi) using identical camera settings and lighting conditions. Images were processed using EOS Utility V.3 software with AxioVision LE64 for precision scale bar generation and calibration. The stereomicroscope’s variable magnification was adjusted appropriately for each species size range to optimize image quality and measurement accuracy.

Frond length (maximum distance from apex to base), frond width (maximum width perpendicular to the longitudinal axis), and length-to-width ratio were measured using ImageJ software v1.8.0_172 [50]. To ensure consistency and robustness, non-parametric statistical tests were employed: Wilcoxon rank-sum tests for two-group comparisons (*S. polyrhiza* and *W. globosa*) and Kruskal–Wallis tests for three-group comparisons (*Le. aequinoctialis*). The significance threshold was set at α = 0.05. Data visualization included boxplots for trait distributions and scatter plots for examining relationships between frond dimensions across genetic clusters. All analyses were conducted using R software (version 4.1.2) [42].

## 5. Conclusions

This study provides a comprehensive assessment of the genetic diversity and population structure of Thai duckweeds by integrating morphological data with molecular analysis using three chloroplast gene markers: *rbcL*, *atpF-H*, and *psbK-I.* Four duckweed species were verified in Thailand: *La. punctata*, *Le. aequinoctialis*, *S. polyrhiza*, and *W. globosa.* These species exhibited varying levels of intraspecific genetic differentiation. *Lemna aequinoctialis* displayed the most complex population structure with four distinct clusters, while *S. polyrhiza* showed moderate diversity with three clusters. *Wolffia globosa* had the highest nucleotide diversity but only two genetic clusters. In contrast, *La. punctata* exhibited the lowest genetic diversity and maintained a highly uniform genetic background across all regions of Thailand.

Geographically, the central region of Thailand—particularly Bangkok and neighboring provinces—emerged as a hotspot of genetic diversity, especially for *Le. aequinoctialis* and *S. polyrhiza*. Significant correlations between genetic clusters and frond morphology further highlight the influence of genetic variation on phenotypic traits. Altogether, these findings enhance our understanding of duckweed biodiversity in Thailand and provide a valuable foundation for the potential use of locally adapted duckweed strains for future applications.

## Figures and Tables

**Table 1:** Summary of genetic diversity and neutrality test statistics for four duckweed species based on concatenated chloroplast sequences (*rbcL*, *atpF-H*, and *psbK-I*).

Species	Sample Size	Length (bp)	GC Content (%)	Haplotype Diversity (h)	Segregating Sites (S)	Nucleotide Diversity (π)	Tajima’s D	*p*-Value
*Landoltia punctata*	5	1475	33.40%	1.000	152	0.038	−1.625	0.104
*Lemna aequinoctialis*	16	1434	33.56%	1.000	502	0.048	−2.276	0.023 *
*Spirodela polyrhiza*	10	1484	33.19%	1.000	452	0.032	−3.344	0.001 *
*Wolffia globosa*	7	1340	34.00%	1.000	418	0.099	−1.271	0.204

* Indicates a significant difference from the assumption that D follows a normal distribution with mean zero and variance one, equivalent to a z-test.

## Data Availability

Newly generated DNA sequences are deposited in the Nucleotide Database at the National Center for Biotechnology Information.

## Supplementary Materials

The following supporting information can be downloaded at: https://www.mdpi.com/article/10.3390/plants14132030/s1, Table S1: Maximum likelihood analysis of *rbcL* gene sequences of four duckweed genera (*Landoltia*, *Lemna*, *Spirodela*, and *Wolffia*) for phylogenetic reconstruction; Table S2: Morphological characteristics and statistical analysis of three duckweed species among genetic clusters; Table S3: Locations and GenBank accession numbers of new specimens collected for the current study; Table S4: Primers used for amplification of chloroplast DNA regions; Table S5: *rbcL* accession numbers of duckweed samples used for phylogenetic study.
