# Supplementary material for: Genetic and Morphological Variation Among Populations of Duckweed Species in Thailand
Source: Plants (Basel). 2025 Jul 2;14(13):2030. doi: 10.3390/plants14132030 (PMC12251731; doi:10.3390/plants14132030)
Supplement: Supplementary file 1 [file plants-14-02030-s001.zip › plants-3672824-supplementary.pdf]

Supplementary Materials:

Genetic and morphological variation among populations of duckweed species in Thailand

Table S1 Maximum likelihood analysis of *rbcL* gene sequences of four duckweed genera (*Landoltia, Lemna, Spirodela,* and *Wolffia*) for phylogenetic reconstruction.

Genus	Nucleotide sites (bp)	Constant sites (bp)	Log-likelihood of consensus tree
Landoltia	500	446 (89.20%)	-1046.848160
Lemna	1409	1278 (90.70%)	-3189.166409
Spirodela	497	431 (86.72%)	-1162.396111
Wolffia	543	477 (87.85%)	-1295.291544

Table S2 Morphological characteristics and statistical analysis of three duckweed species among genetic clusters.

Species	Parameter	Genetic clusters	N	Mean ± SD (mm)	Test	Statistical value	p-value
Lemna aequinoctialis	Frond length	L1	10	3.32 ± 0.53	Kruskal-Wallis test	$\chi2$ = 24.858	4.0e-06*
		L2	60	2.99 ± 0.48			
		L3	20	3.78 ± 0.53			
	Frond width	L1	10	2.15 ± 0.36	Kruskal-Wallis test	$\chi2$ = 46.183	9.4e-11*
		L2	60	1.81 ± 0.26			
		L3	20	2.79 ± 0.41			
	Length: width ratio	L1	10	1.55 ± 0.09	Kruskal-Wallis test	$\chi2$ = 37.287	8.0e-09*
		L2	60	1.66 ± 0.20			
		L3	20	1.36 ± 0.08			
Spirodela polyrhiza	Frond length	S1	20	7.37 ± 0.82	Wilcoxon rank-sum test	W= 632.5	0.0003*
		S2	40	6.36 ± 1.16			
	Frond width	S1	20	5.57 ± 1.33	Wilcoxon rank-sum test	W = 425.5	0.6950
		S2	40	5.36 ± 0.96			
	Length: width ratio	S1	20	1.37 ± 0.23	Wilcoxon rank-sum test	W = 568.5	0.0084*
		S2	40	1.19 ± 0.11			
Wolffia globosa	Frond length	W1	30	0.86 ± 0.23	Wilcoxon rank-sum test	W = 139	0.0015*
		W2	20	1.02 ± 0.13			
	Frond width	W1	30	0.64 ± 0.20	Wilcoxon rank-sum test	W = 142	0.0018*
		W2	20	0.81 ± 0.10			
	Length: width ratio	W1	30	1.36 ± 0.10	Wilcoxon rank-sum test	W = 471	0.0007*
		W2	20	1.26 ± 0.09			

* Indicates significant differences among or between genetic clusters ($p < 0.01$)

Table S3 Locations and GenBank accession numbers of new specimens collected for the current study.

Species	Province	Code	Genetic Cluster	Latitude (°N)	Longitude (°E)	Accession Number*		
						rbcL	*atpF-atpH*	*psbK-psbI*
Landoltia punctata	Bangkok	BK1	LA1	13.850	100.573			
	Bangkok	BK13	LA1	13.845	100.571			
	Khon Kaen	KK24	LA1	16.476	102.825			
	Songkhla	SK3	LA1	7.008	100.498			
	Prachuap Khiri Khan	PK13	LA1	12.234	99.928			
Lemna aequinoctialis	Bangkok	BK2	L1	13.850	100.573			
	Bangkok	BK8	L1	13.850	100.575			
	Bangkok	BK10	L4	13.847	100.567			
	Bangkok	BK11	L4	13.844	100.571			
	Bangkok	BK21	L3	13.737	100.474			
	Nakhon Pathom	NP18	L1	13.800	100.324			
	Nakhon Pathom	NP19	L1	13.787	100.278			
	Nakhon Pathom	NP22	L1	13.796	100.315			
	Pathum Thani	PT24	L1	14.033	100.729			
	Nonthaburi	NT19	L3	13.851	100.530			
	Ubon Ratchathani	UB2	L2	15.136	104.889			
	Phetchaburi	PB12	L2	13.046	100.083			
	Songkhla	SK5	L2	7.042	100.504			
	Prachuap Khiri Khan	PK11	L2	12.234	99.928			
	Prachuap Khiri Khan	PK14	L2	12.244	99.923			
	Prachuap Khiri Khan	PK17	L2	12.390	99.986			
Spirodela polyrhiza	Bangkok	BK15	S3	13.845	100.571			
	Bangkok	BK23	S2	13.737	100.474			
	Nakhon Pathom	NP20	S3	13.787	100.278			
	Pathum Thani	PT25	S3	14.033	100.729			
	Ratchaburi	RB26	S2	13.588	99.808			
	Ubon Ratchathani	UB1	S1	15.136	104.889			
	Chumphon	CP16	S2	10.542	99.196			
	Songkhla	SK4	S2	7.008	100.498			
	Prachuap Khiri Khan	PK8	S1	12.214	99.882			
	Prachuap Khiri Khan	PK15	S2	12.214	99.882			
Wolffia globosa	Bangkok	BK9	W2	13.850	100.575			
	Bangkok	BK12	W2	13.845	100.571			

Species	Province	Code	Genetic Cluster	Latitude (°N)	Longitude (°E)	Accession Number*		
						rbcL	*atpF-atpH*	*psbK-psbI*
	Nakhon Pathom	NP21	W2	13.787	100.278			
	Ratchaburi	RB25	W1	13.588	99.808			
	Ubon Ratchathani	UB28	W2	15.229	104.907			
	Prachuap Khiri Khan	PK7	W1	12.390	99.986			
	Prachuap Khiri Khan	PK10	W1	12.234	99.928			

* will be available at the time of acceptance

Table S4 Primers used for amplification of chloroplast DNA regions.

Region	Direction	Primer Sequence (5' - 3')
rbcL	Forward	GCAGCATTTCGRATGACTC
	Reverse	CTCATTACGGGCTTGTACAC
atpF-atpH	Forward	ACTCGCACACACTCCCTTTCC
	Reverse	GCTTTTATGGAAGCTTTAACAAT
psbK-psbI	Forward	TTAGCATTTGTTTGGCAAG
	Reverse	AAAGTTTGAGAGTAAGCAT

Table S5 *rbcL* accession numbers of duckweed samples used for phylogenetic study.

Genus	Species	Accession number
Landoltia	*La. punctata*	AY034223
Lemna	*Le. trisulca*	AY034237
	Le. minuta	AY034224
	Le. disperma	AY034236
	Le. gibba	AY034235
	Le. minor	AY034234
	Le. japonica	AY034233
	Le. obscura	AY034232
	Le. turionifera	AY034230
	Le. perpusilla	AY034229
	Le. aequinoctialis	AY034228
	Le. tenera	AY034227
	Le. valdiviana	AY034225
Spirodela	*S. polyrhiza*	AM905731
	S. intermedia	SIU68092
Wolffia	*W. globosa*	AY034257
	W. arrhiza	AY034254
	W. brasiliensis	AY034248
	W. microscopica	AY034249
	W. australiana	AY034251

Genus	Species	Accession number
	W. columbiana	AY034255
	W. angusta	AY034253
	W. borealis	AY034250
	W. cylindracea	AY034256
	W. neglecta	AY034252
	W. elongata	AY034258
